# Effects of obesity on sGCβ_1_ mediated signaling in white adipose tissue

**DOI:** 10.1186/2050-6511-16-S1-A83

**Published:** 2015-09-02

**Authors:** Abhishek Sanyal, Linda Sarah Hoffmann, Jennifer Etzrodt, Alexander Pfeifer

**Affiliations:** 1 Institut für Pharmakologie und Toxikologie, Universität Bonn, Germany

## Background

Obesity has reached pandemic proportions with 1.9 billion overweight adults; 600 million of whom are obese [1]. It has been shown that stimulating cyclic 3’-5’ guanosine monophosphate (cGMP) signaling is able to promote adipose tissue homeostasis in obese mice [2]. Conversely, genetic depletion of cGMP dependent protein kinase 1 (PKG1), causes hepatic inflammation and fasting hyperglycemia in mice [3]. Furthermore, cGMP signaling is essential for differentiation of preadipocytes to mature adipocytes *in vitro* [4,5]. PKG1 activation blocks RhoA mediated inhibition of insulin receptor substrate 1 in adipocytes *in vitro* [4,5], thus maintaining insulin sensitivity. The status of cGMP signaling cascade in adipose tissue under obese conditions is not yet fully understood. Here, we examine the functional status of cGMP signaling in white adipose tissue (WAT) of lean and obese mice.

## Materials and methods

For the purposes of this study, WAT from genetically (*ob/ob*) and diet induced obese (DIO) mice was compared to their respective lean controls. Western blot (WB) and qPCR analyses were preformed to ascertain expression levels of cGMP signaling cascade components: soluble guanylyl cyclase β_1_ subunit (sGCβ_1_) and PKG1. Tissue cGMP levels were also measured as an indicator of sGC function.

## Results

Protein expression of sGCβ_1_ was reduced by 50% in WAT of obese mice as compared to their respective lean controls. Similarly, sGCβ_1_ mRNA was also decreased by approximately 50%. In line, cGMP production induced by NO donor (10µM S-Nitroso-N-acetyl-DL-penicillamine) stimulation was a third in WAT of *ob/ob* mice compared to *ob/+*. PKG1 protein and mRNA expression was halved in the WAT of obese mice (both *ob/ob* and DIO) as compared to controls.

## Conclusion

Results thus far indicate that key components of the cGMP cascade (sGCβ_1_ and PKG1) are down-regulated in WAT of obese mice. As a consequence of sGCβ_1_ repression, cGMP production is also dented. Considering the importance of cGMP signaling in maintaining adipocyte function and tissue homeostasis [2-5], disruption of the cascade in WAT could result in the co-morbidities associated with obesity, like hyperlipidemia and insulin resistance. Hampered preadipocyte differentiation due to cGMP signaling dysfunction could result in incapacitated adipocytes leading to ectopic lipid deposition, exacerbating tissue homeostasis. Retardation of the cGMP cascade could result from ER stress and/ or inflammation, which are escalated in obesity [6,7]. It is therefore, essential to delineate the causes for cGMP cascade disruption in this context. Furthermore, targeting the cGMP cascade could help normalize adipocyte function and tissue homeostasis in obese WAT.
